# Direct Biodiesel Production from Wet Microalgae Biomass of *Chlorella pyrenoidosa* through *In Situ* Transesterification

**DOI:** 10.1155/2013/930686

**Published:** 2013-09-30

**Authors:** Hechun Cao, Zhiling Zhang, Xuwen Wu, Xiaoling Miao

**Affiliations:** ^1^State Key Laboratory of Microbial Metabolism, Shanghai Jiao Tong University, Shanghai 200240, China; ^2^School of Life Science and Biotechnology, Shanghai Jiao Tong University, Shanghai 200240, China

## Abstract

A one-step process was applied to directly converting wet oil-bearing microalgae biomass of *Chlorella pyrenoidosa* containing about 90% of water into biodiesel. In order to investigate the effects of water content on biodiesel production, distilled water was added to dried microalgae biomass to form wet biomass used to produce biodiesel. The results showed that at lower temperature of 90°C, water had a negative effect on biodiesel production. The biodiesel yield decreased from 91.4% to 10.3% as water content increased from 0% to 90%. Higher temperature could compensate the negative effect. When temperature reached 150°C, there was no negative effect, and biodiesel yield was over 100%. Based on the above research, wet microalgae biomass was directly applied to biodiesel production, and the optimal conditions were investigated. Under the optimal conditions of 100 mg dry weight equivalent wet microalgae biomass, 4 mL methanol, 8 mL n-hexane, 0.5 M H_2_SO_4_, 120°C, and 180 min reaction time, the biodiesel yield reached as high as 92.5% and the FAME content was 93.2%. The results suggested that biodiesel could be effectively produced directly from wet microalgae biomass and this effort may offer the benefits of energy requirements for biodiesel production.

## 1. Introduction

An everlasting consumption of fossil fuels and effect of greenhouse-gas emissions on global climate change, the world is compelled to focus on finding alternative fuels to the existing fossil fuels [1]. Biodiesel, as an alternative fuel, is considered as one of the most promising alternatives. A large number of studies have shown that biodiesel is environment-friendly, renewable, and biodegradable. Moreover, compared to traditional fossil fuels, biodiesel has lower CO, CO_2_, and hydrocarbon emissions [1–5]. Microalgae, which embody lots of unparalleled advantages as nonfood resources, are viewed to be a promising feedstock of the third-generation biodiesel [6–11]. 

Traditionally, biodiesel production from wet microalgae biomass is through a series of steps including preparation of dry algal powder, extraction of algal oil using organic solvents (such as methanol and chloroform), and conversion of algal oil to biodiesel in the presence of alcohol (methanol or ethanol) and catalyst (acid or base) under certain conditions [12]. Although microalgae-biodiesel has drawn a lot of interest from the economical and environmental sectors, the high production cost is a major obstacle for successful commercial production. A recent life cycle assessment (LCA) of biodiesel production from *Chlorella vulgaris* indicated that drying and algal oil extraction accounted for up to 90% of the total process energy cost [13]. These data suggest that drying microalgae biomass and treating it as a resource for solvent extraction and subsequent transesterification processes would not likely be a net energy positive route toward sustainable biodiesel production. Therefore, any biodiesel production process that obviates biomass drying and organic solvents use for oil extraction could lead to significant energy and cost savings. *In situ* transesterification is considered as an effective approach to avert oil extraction and directly convert the lipids within microalgae cells into biodiesel in a single-step, which not only could simplify the processes of biodiesel production, but also could produce more biodiesel [14]. According to a LCA [13], to produce 1 Kg biodiesel using dried microalgae biomass, the total energy balance was −2.6 MJ, but when using wet biomass, the energy balance was 105 MJ. It is necessary and emergent to use wet microalgae biomass directly as feedstock to produce biodiesel and make microalgae-biodiesel economically and sustainably. Unfortunately, previous attempts of directly producing biodiesel from wet-microalgae have encountered limited success.

In this work, we aimed to find out the effect of water content on biodiesel production from microalgae and optimize the reaction conditions of biodiesel production directly from wet microalgae biomass. Lyophilized-biomass of *Chlorella pyrenoidosa* added with distilled water was used as feedstock to produce biodiesel by *in situ* transesterification, and then based on these experimental trials, wet biomass of *Chlorella pyrenoidosa *was applied directly to biodiesel production through a one step process.

## 2. Materials and Methods

### 2.1. Strain and Culture Conditions

The green microalga *Chlorella pyrenoidosa* was purchased from the algae strain laboratory of Aquatic Organism Research Institute of Chinese Academy of Science. It was maintained in 700 mL of media in 1 L baffled flasks, illuminated by Cool White fluorescent lighting (40 *μ*mol/(m^2^ s)), and aerated with air from the bottom of the vessel with an aeration flow rate of 200 mL/min (i.e., 0.25 vvm, volume gas per volume media per min). A modified BG11 medium was used as the basic medium which contains 100 mg NaNO_3_, 74.9 mg MgSO_4_·7H_2_O, 30.5 mg KH_2_PO_4_, 27.18 mg CaCl_2_, 20 mg Na_2_CO_3_, 8.9 mg C_6_H_5_O_7_Na_3_·2H_2_O, 6 mg ferric ammonium citrate, 1.04 mg Na_2_·EDTA, 2.86 mg boric acid, 0.222 mg ZnSO_4_·7H_2_O, 0.079 mg CuSO_4_·5H_2_O, 1.81 mg MnCl_2_·4H_2_O, 0.39 mg Na_2_MoO_4_, and 0.049 mg Co(NO_3_)_2_·6H_2_O per liter. Glucose (10 g/L) was used for heterotrophic cultivation of microalgae cells. The details of culture of heterotrophic cells were reported in our previous research [15]. Once the culture reached stationary growth phase, cells were harvested by centrifugation, frozen immediately, and then lyophilized prior to lipid analysis and subsequent* in situ* transesterification. As for biodiesel production directly from wet microalgae biomass, the microalgae biomass was harvested and stored immediately in a refrigerator at −80°C.

### 2.2. Lipids Content

Total lipids in *Chlorella pyrenoidosa* cells were extracted according to a modified method from Xu et al. [16]. Lyophilized-dry microalgae powder (200 mg) was triturated in liquid nitrogen for cell fragmentation then was blended with 3 mL chloroform/methanol (2 : 1), shaken for 20 min and centrifuged (4,500 g) for 10 min. The supernatant was collected in a preweighed vial. The process was repeated twice. All the supernatants of were collected together, evaporated, and dried to constant weight at 45°C. The dried vials were weighed to establish the total lipids.

### 2.3. *In Situ* Transesterification

A method of *in situ* transesterification of microalgae biomass was introduced to produce biodiesel. In order to investigate the effects of water content on biodiesel production, according to the method of Wahlen et al. [14], different volumes of distilled water ranging from 0 to 9 mL were added back to lyophilize-dried *Chlorella pyrenoidosa* biomass to form wet microalgae biomass with different water content from 0 to 90%. The *in situ* transesterification reaction took place in a 20 mL-cylindrical tetrafluoroethylene reaction vessel covered with a steel shell to make the reactor air-proof and be safe from high temperature. When using dried biomass added with distilled water to produce biodiesel, 1 g microalgae powder with different volumes (0–9 mL) of distilled water and three different temperatures of 90, 120, and 150°C were used. The optimal reaction conditions (4 mL methanol, 6 mL n-hexane, 0.5 M H_2_SO_4_, 120 min) reported in our previous study were applied to this system [15]. When directly using wet biomass to produce biodiesel, 100 mg dry weight equivalent wet microalgae biomass was used. Three different temperatures (90, 120, and 150°C), five different reaction times (90, 120, 180, 240 and 300 min), five levels of methanol volume (1, 2, 4, 6, 8 mL), and five levels of n-hexane volume (2, 4, 6, 8, 10 mL) were used for the experimental trials to establish optimal conditions.

 After the reaction stopped, 2.0 mL distilled water was added to the mixture. Samples were then cooled down to room temperature, centrifuged at 8,228 g for 10 min. Three layers formed which were composed of water phase (lower layer) containing water and alcohol, solid phase (middle layer) containing microalgae residue, and organic phase (upper layer) containing solvent and biodiesel, respectively. The organic layer was collected and evaporated at 45°C to constant weight for analysis. The biodiesel yield from microalgae biomass was calculated by (1)

(1) Biodiesel  yield  (%)  =Biodiesel  mass(g)algae  mass(g)×oil  content(%)×100%.

### 2.4. Analysis of Biodiesel Composition

The composition of biodiesel produced from *in situ* transesterification of microalgae biomass was analyzed by GC-linked mass spectrometry (GC-MS) equipped with a DB-5MS column (30 m × 0.25 mm ID DF = 0.25 *μ*m) and with a flow rate of 1.0 mL/min.

## 3. Results and Discussion

### 3.1. Effect of Water Content and Temperature on Microalgae Biodiesel Production

**Figure 1:**
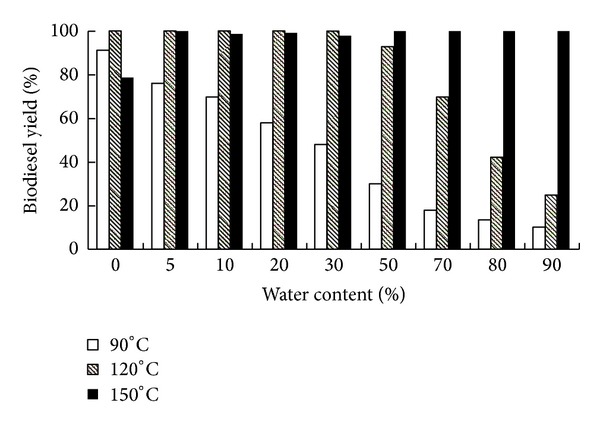
Effects of water content and temperature on biodiesel yield by *in situ *transesterification of dried *Chlorella pyrenoidosa* biomass with distilled water. Reaction conditions: 1 g dried microalgae biomass (oil content 47%) with 4 mL methanol, 6 mL n-hexane and 0.5 M sulfuric acid for 120 min. Water content ranged from 0 to 90%. Temperature: 90, 120, and 150°C.

Before directly using wet microalgae biomass as feedstock to produce biodiesel, the effects of water content and temperature on biodiesel production from dried microalgae biomass with distilled water were studied. The results were shown in Figure 1. From the results obtained (Figure 1), it was observed that biodiesel yield decreased while water content increased from 0 to 90% at lower temperature of 90°C. The biodiesel yield was 91.4% when water content was 0%, and when water content was increased to 90%, the biodiesel yield was only 10.3% (Figure 1). This was in accordance with the previous study that water could dramatically impede biodiesel production [14]. However, at higher temperature of 120°C, lower water content from 0% to 30% seemed to have no effects on biodiesel yield. The biodiesel yields were all over 100%. This was probably because other molecules (e.g., phospholipids) were also converted into biodiesel [14]. Further, increasing the water content from 50% to 90%, the biodiesel yields began to decrease, which was only 24.8% under 90% water content (Figure 1). The FAME contents of the biodiesels from dried microalgae biomass with different water content under different temperatures are shown in Table 1. As shown in Table 1, the FAME contents were all over 87% under different water content at temperature of 120°C. These results suggested that higher temperature could partially compensate the negative effect of water on transesterification. This is probably because high temperature could speed up the reaction (microalgae biomass, methanol, n-hexane, and catalyst) and make transesterification reaction take place more effectively. Available water requires more energy to cause the reaction to effectively occur. Wahlen et al. [14] found that when using 100 mg microalgae biomass with 400 mg distilled water (water content 80%) to produce biodiesel, the FAME yield was 54% of the expected under the reaction conditions of 5 mL methanol, 1.8% (*v*/*v*) H_2_SO_4_, 80°C, and 20 min of reaction time. In our study, when 9 mL distilled water was added back to 1 g microalgae biomass (water content 90%), biodiesel yield was over 100% (Figure 1) and FAME content was 89.81% (Table 1) at temperature of 150°C, more biodiesel produced than expected. As shown in Figure 1, the biodiesel yields were all over 98% under the water content ranging from 5% to 90% at temperature of 150°C, and the FAME content was almost all over 88% (Table 1). It was reasonable that available excess water compensated for the effects higher temperature in the reaction mixture.

### 3.2. Effects of Temperature and Reaction Time on Biodiesel Production from Wet Biomass 

**Figure 2:**
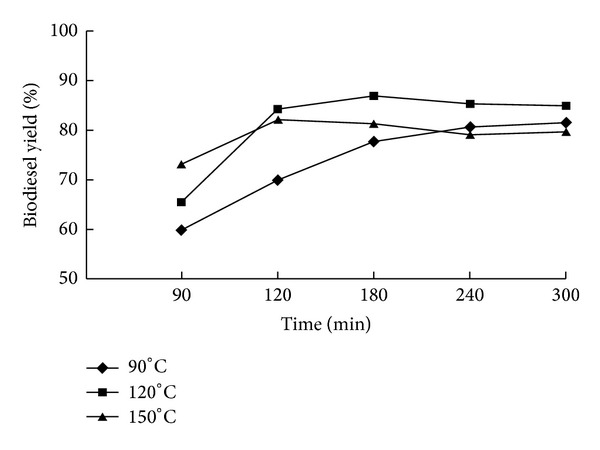
Effects of reaction time and temperature on biodiesel yield by *in situ *transesterification of wet *Chlorella pyrenoidosa* biomass. Reaction conditions: 100 mg dry weight equivalent wet biomass (oil content 47%, water content 90%) with 4 mL methanol, 6 mL n-hexane, and 0.5 M sulfuric acid. Reaction time: 90, 120, 180, 240 and 300 min. Temperature: 90, 120, and 150°C.

Based on the above experimental trials, the wet microalgae biomass was directly applied to the biodiesel production. The results of effects of temperature and reaction time on biodiesel production directly from wet microalgae biomass are shown in Figure 2. It suggested that for the samples investigated at 90°C, asymptotic biodiesel conversion values were not reached within the time boundaries of this study. When *in situ* transesterification process was carried out at 120°C, the highest biodiesel yield of 86.8% was obtained within 180 min (Figure 2), the FAME content was about 92% (Table 2). The biodiesel yield was slightly decreased to 85.3% and 84.7% when the reaction time increased to 240 and 300 min, respectively (Figure 2). The FAME contents of biodiesels produced at reaction times of 240 and 300 min under 120°C were all about 90% (Table 2). These results suggest that longer reaction time could not efficiently produce more biodiesel. As reported by Patil et al. [17], extended reaction time may result in overheating of the reaction mixture, greater losses of solvent, by-product formation, and energy losses. As shown in Figure 2, the highest biodiesel yield of 82.1% was obtained within 120 min at temperature of 150°C, and FAME content was 90.56% (Table 2). The biodiesel yield was slightly decreased as the reaction time was increased (Figure 2). The results were in accordance with previous studies that with temperature rising, the time to reach highest biodiesel yield was shortened [14, 15, 18, 19]. When dried microalgae biomass was used as feedstock to produce biodiesel, 90°C or lower temperature was preferred [15, 18]. When wet microalgae biomass was directly used as feedstock to produce biodiesel, 120°C was preferred. This may be because that water within microalgae cell protected the cell from destroying and impeding the contact of methanol with lipids. Higher temperature could improve cell disruption and make the contact of methanol with lipids much easier, and result in a higher biodiesel yield. However, when further increasing temperature to 150°C, it was found that less biodiesel was produced as compared to that at 120°C. This was probably because that 150°C was too high to burn some of the oil [15, 20].

We assumed that the reaction process of *in situ* transesterification followed the following steps: (1) microalgae cells were destroyed or partially destroyed; (2) lipids within microalgae cells were released outside through cells wall; (3) the lipids contacted with reactants; (4) reaction took place. The solvent (n-hexane) added was of importance because the contact of algal lipids with reactant was the vital step in transesterification reaction and the addition of n-hexane could help lipids within microalgae cells be easily extracted. As reported in our previous study [15], n-hexane was an optimal solvent because the biodiesel yield could be dramatically improved when n-hexane was added. Beside, n-hexane is commonly used for oil extraction, and it has been demonstrated that more lipids could be easily extracted from microalgae cells by using n-hexane as an extraction solvent [1, 21, 22].

### 3.3. Optimizing Conditions of Biodiesel Production from Wet Microalgae Biomass

**Figure 3:**
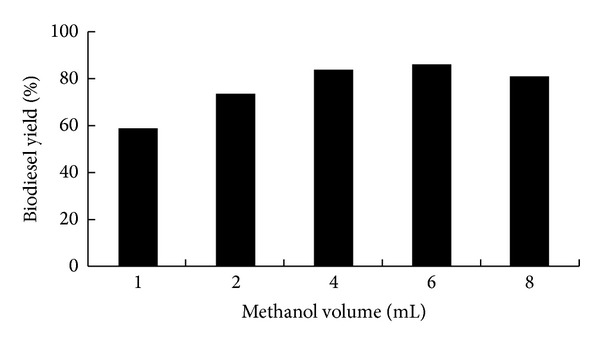
Effects of methanol volume on the biodiesel yield by *in situ *transesterification of wet *Chlorella pyrenoidosa* biomass. Reaction conditions: 100 mg dry weight equivalent wet biomass (oil content 47%, water content 90%) with 6 mL n-hexane and 0.5 M sulfuric acid at 120°C for 120 min. Methanol volume: 1, 2, 4, 6, and 8 mL.

 Methanol always played an important role in transesterification because during the transesterification reaction, methanol was considered not only as reactant, converting the lipids to fatty acid alkyl esters, but also as solvent, extracting the lipids from the biomass. Previous studies have demonstrated that methanol was optimal for biodiesel production [14, 23] and the volume of methanol had a dramatic effect on biodiesel yield [14, 15, 24–26]. To identify the optimal methanol volume, wet microalgae biomass (100 mg dry weight equivalent) of *Chlorella pyrenoidosa* were reacted with 6 mL n-hexane, 0.5 M sulfuric acid and different volumes of methanol (1, 2, 4, 6, and 8 mL) at 120°C for 120 min. The results showed that the biodiesel yield was increased from 59.2% to 83.9% with the methanol volume increasing from 1 mL to 4 mL (Figure 3). The maximum biodiesel yield of 86.6% was obtained at 6 mL of methanol. Further, increasing methanol volume to 8 mL, the biodiesel yield was decreased to 81.3%.

Wahlen et al. [14] determined that to reach the maximum biodiesel productivity, 2.5 mL methanol was needed for 100 mg dried microalgae biomass. Patil et al. [17] suggested that during transesterification, more methanol was needed to shift the reversible reaction forward (as observed) perhaps due to the increased contact area between methanol and lipid, resulting in higher yield of FAME. In the present research, we found that the volume of methanol necessary for maximal direct conversion of algal lipids to biodiesel was higher than that reported by Wahlen et al. [14]. More methanol was needed for optimal direct conversion of lipids from wet microalgae biomass, which is probably because the water within cells may dilute the methanol and make the contact of lipids with methanol much harder, although the maximum biodiesel yield was reached at level of 6 mL methanol, which was only 2.7% higher than that at level, of 4 mL methanol. From the practical point of view, it was beneficial to keep the methanol levels as low as possible to reduce the downstream separation costs. Therefore, 4 mL methanol was selected for the following study. 

**Figure 4:**
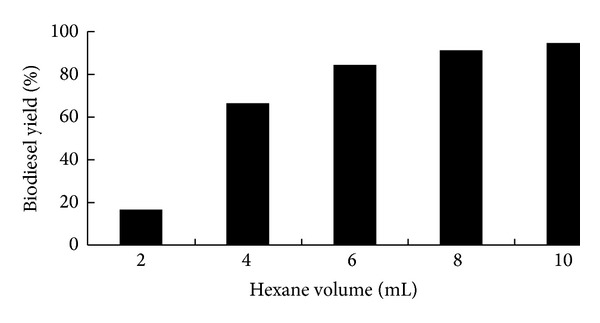
Effects of n-hexane volume on the biodiesel yield by *in situ *transesterification of wet *Chlorella pyrenoidosa* biomass. Reaction conditions: 100 mg dry weight equivalent wet biomass (oil content 47%, water content 90%) with 4 mL methanol and 0.5 M sulfuric acid at 120°C for 120 min. n-Hexane volume: 2, 4, 6, 8, and 10 mL.

In our previous study [15], we found that when other extra solvent especially n-hexane was added to the reaction system, the biodiesel yield could be dramatically increased. It may be because that when n-hexane was added, it could make the contact of methanol with lipids within cells much easier, and thus more biodiesel was produced. To identify the optimal amount of n-hexane during *in situ* transesterification directly using wet microalgae biomass as feedstock, wet samples (100 mg dry weight equivalent) of *Chlorella pyrenoidosa* were incubated with 4 mL methanol, 0.5 M sulfuric acid, and different volumes of n-hexane (2, 4, 6, 8, and 10 mL) at 120°C for 120 min. The effect of n-hexane on biodiesel yield was shown in Figure 4. The biodiesel yield was dramatically increased from 16.6% to 84.0% when n-hexane was increased from 2 mL to 6 mL. This was in accordance with our previous study that the high biodiesel yield could be obtained under high volume of n-hexane [15]. The biodiesel yields were 90.9% and 94.5% at n-hexane volumes of 8 and 10 mL, respectively (Figure 4). On economic basis, 8 mL n-hexane was chosen. Then, the optimized conditions of 100 mg dry weight equivalent wet microalgae biomass, 4 mL methanol, 8 mL n-hexane, 0.5 M H_2_SO_4_ with temperature of 120°C, and 180 min reaction time were selected.

### 3.4. Biodiesel Yield from Wet Microalgae under Optimal Conditions

The optimal conditions (4 mL methanol, 8 mL n-hexane, 0.5 M H_2_SO_4_ with temperature of 120°C, and 180 min reaction time) were applied to *in situ* transesterification of wet microalgae biomass for biodiesel production. The biodiesel yield could reach as high as 92.5% and FAME content was 93.2%.

## 4. Conclusions

Water could generate a significant negative effect on *in situ* biodiesel production when dried microalgae biomass suspended in distilled water was used as feedstock at low temperature. However, this negative effect could be compensated by higher temperature. High biodiesel yield and high quality biodiesel could be obtained through *in situ* transesterification directly from wet microalgae biomass. This effort may offer the benefits of energy requirements for biodiesel production by eliminating the needs for drying and extraction of microalgae biomass. The *in situ* transesterification of wet microalgae biomass had the potential to provide an energy efficient and economical route to microalgae biodiesel production.

## Figures and Tables

**Table 1:** FAME contents of the biodiesels from dried *Chlorella pyrenoidosa* biomass with different water content under different temperature through *in situ *transesterification. Reaction conditions: 1 g microalgae biomass (oil content 47%) with 4 mL methanol, 6 mL n-hexane, and 0.5 M sulfuric acid for 120 min.

Water content (%) (g/g)	FAME content (%)		
	90°C	120°C	150°C
0	93.24	91.08	90.89
5	90.38	90.48	89.12
10	88.72	89.57	88.72
20	89.46	90.38	92.17
30	91.75	88.58	91.58
50	86.47	89.76	87.31
70	89.13	87.48	88.68
80	88.91	89.69	90.26
90	90.25	87.73	89.81

**Table 2:** FAME contents of the biodiesels from wet *Chlorella pyrenoidosa* biomass under different temperature and different reaction time by *in situ *transesterification. Reaction conditions: 100 mg dry weight equivalent wet biomass (oil content 47%, water content 90%) with 4 mL methanol, 6 mL n-hexane, and 0.5 M sulfuric acid.

Reaction time (min)	FAME content (%)		
	90°C	120°C	150°C
90	90.76	92.89	89.75
120	87.63	91.78	90.56
180	90.14	92.14	90.17
240	89.39	90.27	88.26
300	90.57	89.85	87.92
